# Digital health and COVID-19

**DOI:** 10.2471/BLT.20.021120

**Published:** 2020-11-01

## Abstract

The novel coronavirus pandemic is increasing demand for digital health in primary care delivery, highlighting the progress being made and the challenges still faced. Gary Humphreys reports.

When Sri Lanka began to report its first confirmed novel coronavirus disease (COVID-19) at the beginning of the year, the Ministry of Health (MOH) put into action a response plan that included one of the newest tools in its tool kit: digital health.

“The first case of COVID-19 was reported in the last week of January, but the outbreak gained momentum in mid-March,” says Professor Vajira Dissanayake, Dean of the Faculty of Medicine at Colombo University in Sri Lanka, and one of the drivers of Sri Lanka’s efforts to build digital health capacity over the past 20 years. “There was a concern that the health system might be overwhelmed, so people were encouraged to get information and advice over the phone or via the internet.”

A media campaign was launched encouraging people to use a hotline. People who were waiting in line at designated receiving centres for suspected COVID-19 patients were also told about the hotline and encouraged to use it for teleconsultation and for the scheduling of future appointments with doctors.

Several apps were also rolled out, among them MyHealth Sri Lanka, an app launched by the MOH together with the Information and Communication Technology Agency of Sri Lanka. In March, when concern about the health system being overwhelmed was at its peak, the app directed people to private sector telehealth providers that were instructed by the government to provide teleconsultation free of charge.

“As of mid-September we have had around 3500 COVID-19 cases,” Dissanayake says. “Effective use of digital tools has played an important role in keeping that number low.”

It has long been recognized that digital technology has the potential to transform health service delivery, including the delivery of primary health care (PHC). That potential is currently being tested in pandemic responses worldwide, mainly in high-income countries such as Singapore – where recent digital health initiatives include the installation of interactive kiosks in migrant workers’ dormitories – but also in low- and middle-income countries, Sri Lanka being one example.

“Some countries have accelerated shifts in models of care in response to the pandemic, while others are making use of available infrastructure to deliver essential health services in primary health-care settings,” says Diana Zandi, a technical officer working on digital health and primary health care at the World Health Organization (WHO).

Teleconsultation (remote consultation using information and communications technologies) is being used to reduce health workers’ exposure to the novel coronavirus, while also taking pressure off health systems.

“Countries have accelerated shifts in models of care in response to the pandemic.”

Diana Zandi

In the Republic of Indonesia, for example, the MOH is supporting and promoting the use of telehealth services, and the President of Indonesia has encouraged the public to access online services, whether for regular check-ups or for COVID-19 related enquiries.

According to Anis Fuad, a public health researcher at the Gadjah Mada University in Yogyakarta, Indonesia, the MOH, working in collaboration with the social security agency, BPJS Kesehatan, entered into agreements with 20 telehealth firms in the first quarter of 2020, making it possible for patients to get information and guidance or to be connected with doctors for consultations via text, telephone and video.

The MOH also created a COVID-19 feature on its SehatPedia app, an interactive health information app which it launched in February 2019. The COVID-19 feature provides information regarding the transmission and health risks posed by the virus as well as a self-assessment tool. It also enables users to directly consult with doctors regarding COVID-19 symptoms. To facilitate access to the providers, the MOH set up a digital call centre in March 2020.

“Collaboration between the MOH, BPJS and health-tech start-ups is likely to bring about a significant increase in the volume and distribution of digital health initiatives in the country,” says Fuad.

Whether those users end up being satisfied with the service they receive will depend on the accessibility and quality of those services.

“Weak primary health-care systems don’t suddenly become strong because you introduce a teleconsultation app,” says Robert Marten, a scientist at the Alliance for Health Policy and Systems Research at WHO, sounding a note of caution. “A country with three doctors per 10 000 people will continue to struggle to meet health care needs, with or without digital health.”

The digital divide – the gap between communities that have the resources required to access digital health services and those that don’t – is another matter of concern. Disparities in digital literacy and access to equipment, broadband and the internet will only become more important as digital health becomes more prevalent.

“The digital divide is not an argument against using digital health,” says Marten, “but it is an argument for ensuring optimal access to services through low-cost, high-impact digital health interventions.”

Such interventions are already being implemented. In Liberia, for example, mobile phones are being used to connect community health workers with the MOH as part of a commitment to making primary health care universal.

The connection is made using a software platform called mHero, which was created by IntraHealth International – a nongovernmental organization committed to health system strengthening – working with the United Nations Children’s Fund, the United States Agency for International Development and the MOH. The platform was set up in August 2014 to support health-sector communication during Liberia’s Ebola outbreak.

“The health ministry’s paper-based reporting system was being overwhelmed,” explains Dana Acciavatti, IntraHealth’s Senior Portfolio Manager. “So, the IntraHealth team, which had recently been in Liberia working on a health workforce information platform, put their heads together with the Ebola Task Force to come up with a solution that allowed the MOH to reach Liberian frontline health workers using basic mobile phones.”

Starting in 2018 and supported by Last Mile Health, a nongovernmental organization that was founded by survivors of Liberia’s civil war, the MOH equipped all community health workers in the programme with mobile equipment to enable data collection and disseminate educational videos to support PHC delivery in remote communities.

“We have equipped 3777 community health workers with a phone, a solar panel for charging, and a battery,” says Geoffrey Mimano, Digital Health Director at Last Mile Health. “This means they can remain operational even in situations where there is no power grid. The system even works where there is no cell phone network.”

This clever trick is achieved by customizing an open-source digital health platform to allow for offline information transfer via Bluetooth. “Community health supervisors go between the villages gathering data from community health workers, then return to their nearest health facility to upload the data to a central repository,” Mimano explains.

Both Acciavatti and Mimano acknowledge the importance of the MOH’s support in advancing the programme. “Having really strong engagement by the MOH team in Liberia from day one is why it has been so successful there,” Acciavatti says.

Dissanayake also emphasises the importance of government in driving the digital health agenda in Sri Lanka, while highlighting the contribution made by doctors, notably those trained at the Health Informatics Society of Sri Lanka, which was established in 1997 and started offering formal training to doctors in the form of a master’s degree course in biomedical informatics in 2008.

“I think this is an important turning point.”

Lasantha Ranwala

“Those doctors went back to the health ministry once they had completed their studies to inform digital health policy development. Their contribution has been crucial,” Dissanayake says.

Today Sri Lanka’s health ministry employees are building the country’s digital health future – people such as Dr Lasantha Ranwala, Senior Registrar in Health Informatics at the health ministry’s Health Information Unit, who is working on a national personal health record portal and app to support patient-centred care.

“The pandemic has definitely increased the impetus for the deployment of digital health, notably in terms of general public awareness of what digital health has to offer,” Ranwala says. “I think this is an important turning point.”

For WHO’s Zandi, it is vital that governments continue to develop and implement robust, context-specific national strategies, working closely with the private sector to make sure that the patient data collected as a result of digital interactions are properly protected.

“It has to be realized that implementing digital health is about more than simply deploying new technologies or devices,” Zandi says. “It requires system-level thinking and coherent national strategy that comprises effective legislation, regulations and oversight – all of which are needed to ensure quality, safety and security and interoperability of digital health services, including telemedicine.”

**Figure:**
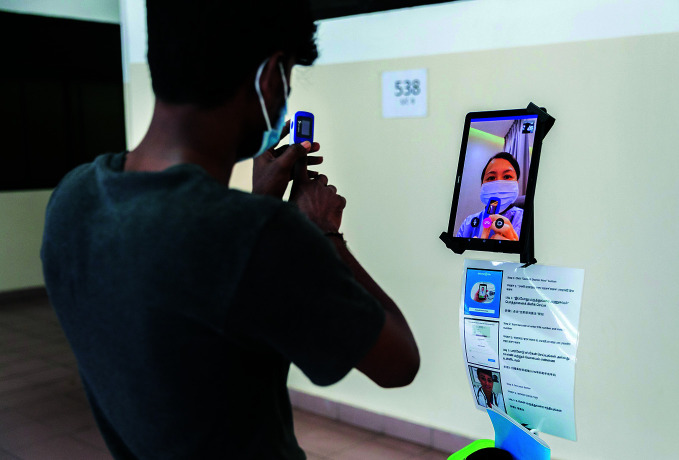
A patient consults a doctor using a tele-kiosk in a migrant worker dormitory in Singapore.

WHO/Juliana Tan

**Figure:**
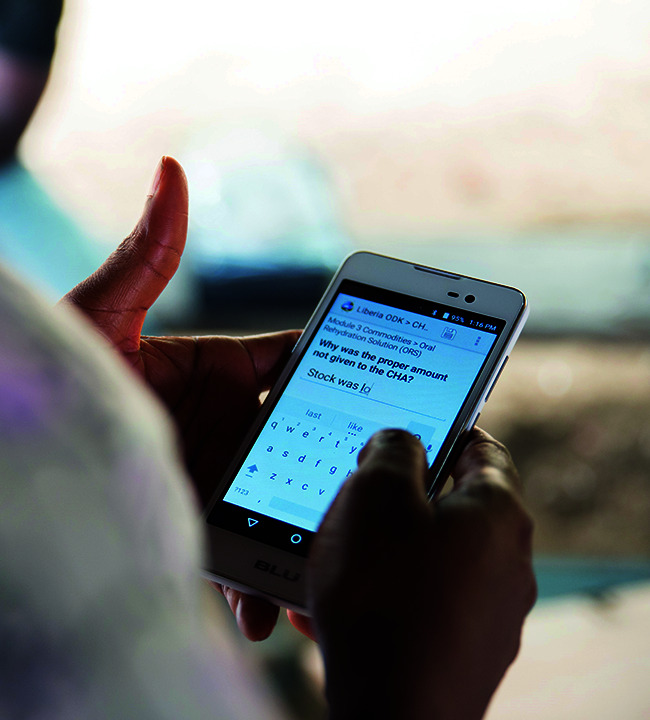
A nurse supervisor in Liberia enters data on a community health worker's essential medicine supplies.

Courtesy of Last Mile Health

